# Difficulties and Coping Strategies of Psychiatric Visiting Nurses After the Noto Peninsula Earthquake: A Qualitative Descriptive Study

**DOI:** 10.3390/nursrep16020047

**Published:** 2026-01-30

**Authors:** Masato Oe, Hisao Nakai, Yutaka Nagayama, Shingo Oe, Chinatsu Yamaguchi, Koji Tanaka

**Affiliations:** 1 Nursing Department, School of Nursing, Kanazawa Medical University, Kanazawa Medical University Hospital, 1-1 Uchinada, Kahoku 920-0265, Japan; naga-y@kanazawa-med.ac.jp (Y.N.); yama-c@kanazawa-med.ac.jp (C.Y.); 2 Kanazawa Medical University, Kanazawa Medical University Hospital, 1-1 Uchinada, Kahoku 920-0265, Japan; 3 Faculty of Nursing, University of Kochi, 2751-1 Ike, Kochi 781-8515, Japan; nakai_hisao@cc.u-kochi.ac.jp; 4 Faculty of Nursing, Ishikawa Prefectural Nursing University, 1-1 Gakuendai, Kahoku 929-1210, Japan; ohe1111@ishikawa-nu.ac.jp; 5 Faculty of Health Sciences, Institute of Medical, Pharmaceutical and Health Sciences, Kanazawa University, 5-11-80 Kodatsuno, Kanazawa 920-0942, Japan; ktanaka@staff.kanazawa-u.ac.jp

**Keywords:** 2024 Noto Peninsula earthquake, psychiatric visiting nurse service, qualitative descriptive study

## Abstract

**Background/Objectives**: The 2024 Noto Peninsula earthquake in Japan severely affected community care for persons with psychiatric disabilities. This study analyzed the difficulties and adaptive coping strategies of psychiatric visiting nurses (PVN) to inform disaster mental health practice. **Methods**: A qualitative, descriptive design was used. Semi-structured interviews were conducted with six PVN, and the data were analyzed thematically. **Results**: Key findings indicated two main challenges: a system-level paralysis of care owing to infrastructure collapse and the ethical dilemmas experienced by the role of PVN as “dual victims.” In response, nurses leveraged pre-existing therapeutic relationships to ensure care continuity and acted as essential liaisons to external teams. The study also documented substantial and unexpected patient resilience. **Conclusions**: Based on the findings, this study’s primary contribution is a recommendation to reframe disaster policy by shifting focus from merely deploying external aid to empowering existing, trusted community care networks and adopting a strengths-based model for mental health support.

## 1. Introduction

On 1 January 2024, a major earthquake measuring 7.6 on the Richter scale occurred in the Noto Peninsula, Ishikawa, Japan, causing extensive damage in the northern part of the peninsula [1,2]. The earthquake triggered massive tectonic movements and a destructive tsunami that hit coastal areas within minutes, causing extensive damage and resulting in 489 deaths [3]. There were an estimated 50,000 evacuees, and over 500 evacuation shelters were set up by the affected local governments [4]. The northern Noto area, which was the epicenter of the earthquake and received the most severe damage, is a geographically isolated region with a population of approximately 16,000 [5]. It has been estimated that in the whole of Ishikawa Prefecture (total population approx. 1.1 million) over 18,000 people received medical treatment for mental disorders in 2022 [6], highlighting the large number of persons with psychiatric disabilities (PPDs) residing in this vulnerable region. The earthquake particularly affected people on the Noto Peninsula who were receiving home care for mental disorders.

Generally, PPDs are more vulnerable to disasters and may be exposed to additional risks during and after a disaster [7,8]. This heightened vulnerability can be conceptualized using frameworks such as the vulnerability-stress model, which posits that major stressors such as natural disasters can exacerbate pre-existing vulnerabilities and lead to adverse mental health outcomes [9,10]. PPDs are at higher risk of injury and death than other groups; additionally, they often have special needs and experience difficulty finding shelter during disasters [11]. Visiting nursing services in Japan are provided to a range of patients, including children and older people. Such services include a psychiatric visiting nurse service (PVNS), which specializes in treating people with mental disorders [12,13]. In the northern part of Noto Peninsula, which was severely affected by the 2024 Noto Peninsula earthquake (NPE), there are three visiting nursing stations specializing in psychiatric care [14].

In 2007, there was a major earthquake in the Noto region [15]; however, the damage caused by the 2024 NPE was much more severe, causing widespread infrastructure collapse and the prolonged isolation of the peninsula. A growing body of research has begun to document the seismological, infrastructural, and general health effects of the 2024 NPE [16,17,18,19,20,21,22]. However, although previous studies on major Japanese disasters have focused on the psychological impact on patients or the challenges for hospital-based staff [23,24], there remains an important gap in the literature; namely, a lack of qualitative research focusing on the on-the-ground difficulties and practical coping strategies of community-based psychiatric visiting nurses (PVN), in the immediate post-disaster phase.

PVN in the affected areas of the Noto region were “dual victims” [25,26] (i.e., both caregivers and survivors), which made the provision of support extremely difficult; furthermore, many PPDs experienced secondary evacuation to distant municipalities [27,28]. The purpose of the present study was to bridge the above-mentioned research gap by qualitatively clarifying the unique difficulties and adaptive coping strategies of PVN after the 2024 NPE. The findings may provide novel and practical insights for psychiatric home nursing to prevent the worsening of PPD symptoms and ensure that they can continue to live in the community. The findings could also be used to improve PVNS provision after disasters, particularly in regions with similar geographical challenges, to plan how to prepare for and manage difficulties during disasters, and to support PPDs who use the PVNS.

## 2. Materials and Methods

### 2.1. Design

This was a qualitative descriptive study. Qualitative research methods can provide a detailed description of a phenomenon, which permits a deeper understanding of the context, experiences, and social phenomena of the people involved [29]. Because the aim of the study was to provide an in-depth description of the difficulties and coping strategies experienced by PVN after the 2024 NPE, we determined that this research method was appropriate. This conduct of this study was guided by the Standards for Reporting Qualitative Research (SRQR) Framework [30].

### 2.2. Study Participants

Study participants were PVN from one visiting nursing station specializing in psychiatry that operated in the Noto Peninsula. All six PVN at this station participated in the study. Because of the disaster, there was a shortage of nursing personnel owing to evacuation of medical workers and a sudden increase in the number of hospitalizations of people with chronic illnesses who could no longer live at home. Therefore, we did not attempt to recruit participants from other nursing stations. The PVNS was affected by the 2024 NPE, and immediately after the disaster began working with external supporters and local healthcare service providers. All the PVN working at this PVNS had experience working on psychiatric wards and providing home visit care. All the selected PVN agreed to participate but only those who provided their consent to participate were included in the study.

### 2.3. Data Collection

To obtain participants who were familiar with northern Japan and the PVNS, we used a purposive sampling method to obtain participants from PVNS stations. Participants were accessed through the administrator of a visiting nurse station in northern Noto. PVN working at this station were asked to participate in the study. Through their previous activities in psychiatric nursing-related organizations, the authors had established relationships with the PVN who took part in the study. Although the authors collaborated with PVN on academic conferences and research, there was no conflict of interest. Data were collected using semi-structured interviews with PVN. An interview guide was used to structure the interview questions. This guide included questions about what kind of support PVN provided after the earthquake, what difficulties they experienced in providing the PVNS, what methods and strategies they used to deal with those difficulties, and how they supported PPDs in their daily lives. Additional questions were included based on participants’ responses. The interviews were conducted by one of the authors who holds a nursing license and a doctoral degree. Each participant was interviewed individually and face to face. All interviews were conducted in private rooms in the nursing station to ensure that participants could talk about their experiences in confidence. The interviews were conducted in July and August 2024, approximately 6 months after the earthquake, and lasted from 30 min to 1 h 19 min (average 46 min). The interviews were recorded verbatim and used as data.

### 2.4. Data Analysis

The verbatim transcripts were read to obtain an overall picture of the data. Next, the data were searched for words and phrases that described content related to home nursing practice in the 2024 NPE, and codes were created at the sentence level. Codes with similar meanings were grouped together at a higher level of abstraction to create sub-themes. Similarly, themes were created by grouping sub-themes at a higher level of abstraction.

During the analysis process, the accuracy of the data was confirmed using Lincoln and Guba’s four criteria: credibility, confirmability, dependability, and transferability [31]. Specifically, to increase credibility, information was collected from the medical records to confirm and facilitate the interpretation of the narrative content. Furthermore, member checks were conducted (i.e., the data were returned to participants for cross-checking) to ensure that the data were accurate and consistent with the research participants’ experiences. During the analysis, we discussed whether there were any errors in the interpretation of the data. Coding, sub-theming, and theming were carried out independently by two of the researchers (M.O. and S.O.). All researchers then reviewed the narrative data that supported the codes, sub-themes, and themes. Where necessary, we amended the names of the codes, sub-themes, and themes. Additionally, the results of the analysis were checked by the study participants to ensure that they were consistent with the participants’ interview data and experiences. The original data were collected and analyzed in Japanese. The findings were translated into English for reporting purposes after content analysis was completed. We carefully reviewed the translations to ensure that the original meaning of the text was preserved. The English text was then translated back into Japanese, and all researchers confirmed that the participants’ narratives and extracted themes were consistent with the original meaning. To increase the reliability of the analysis, two doctoral students (Y.N. and K.T.), who are experts in qualitative research, reviewed the entire analytic process. No software or other tools were used for data analysis.

The participants were six PVN with extensive clinical experience. During the chaotic period following the 2024 NPE, it was difficult to find many nurses to interview, which limited the sample size. However, qualitative research methods are appropriate to investigate small samples, so we considered that the data would be meaningful even with a small number of participants. This decision is supported by research from Guest et al., who demonstrated that data saturation is often reached within the first six to ten interviews, enabling identification of over 90% of relevant themes [32]. Additionally, we had sufficient time to conduct each interview, which allowed us to gather abundant information and obtain in-depth insights. Furthermore, analysis of data from the sixth interview did not yield any new codes or themes relative to the first five interviews. Therefore, we judged that sufficient data saturation had been reached for the purposes of this study, and terminated the analysis.

### 2.5. Ethical Considerations

The study was conducted with the approval of the university medical research ethics review committees at the authors’ universities (No. C093). The participants provided their written informed consent and were informed of the purpose and importance of the study, the method, the fact that participation was voluntary, and the fact that they would not be personally identified when the results were made public.

## 3. Results

Six PVN participated in this study (Table 1). The analysis identified six themes that described the difficulties experienced by PVN and six themes that described their coping strategies (Table 2 and Table 3).

### 3.1. Difficulties Experienced by PVN (Table 2)

#### 3.1.1. Theme 1: Paralysis of Care: The Collapse of Foundational Systems

The most immediate and fundamental challenge participants experienced was an almost total paralysis of care triggered by the collapse of foundational systems. Physical infrastructure problems, such as damage to roads and communication networks, made it impossible for nurses to reach patients, confirm their safety, or coordinate care. This systemic breakdown also severed access to essential medical supplies, resulting in critical disruptions to treatment. One nurse described the struggle to provide medication, a core component of care:

*“Even when the mobile pharmacy came round, specialized psychiatric medicines were not available.”*

*(Participant C)*

This physical isolation was compounded by a communications blackout that left nurses unable to perform even basic safety checks, creating a sense of profound uncertainty. As another participant explained, this severed the most basic link to their patients:

*“We knew their landline numbers but didn’t know their cell phone numbers, so we couldn’t contact them.”*

*(Participant B)*

#### 3.1.2. Theme 2: The “Dual Role” Dilemma: Navigating Personal Trauma and Professional Duty

A major difficulty described by participants was the substantial psychological and ethical conflict arising from their “dual role” as both caregivers and disaster victims. Participants described the dilemma of trying to balance a powerful sense of professional duty to their vulnerable patients and the urgent need to ensure their own safety and that of their families. This created a decision paralysis regarding how to allocate their time and energy. One nurse articulated this core conflict as follows:

*“I don’t know how to prioritize my work as a visiting nurse.”*

*(Participant A)*

This internal conflict was intensified by the direct emotional trauma of witnessing the damage caused by the disaster while trying to provide care. The constant exposure to varying levels of destruction created a considerable emotional burden, making it difficult for the nurses to process their own experiences as survivors. One participant expressed this struggle as follows:

*“It was difficult to sort out my feelings because there was a difference between the areas that were heavily damaged and the areas that were less damaged when I visited.”*

*(Participant D)*

This dual role placed the PVN in a state of continuous conflict, characterized by a tension between their professional identity and their personal reality as victims of the same catastrophe.

#### 3.1.3. Theme 3: Compounded Vulnerability: The Precarious Position of PPDs in a State of Exception

The disaster placed PPDs in a “state of exception,” in which their pre-existing vulnerabilities were severely compounded by the collapse of their routines and the inadequacies of the emergency response systems. Many were caught in a double bind: having to choose between either remaining in dangerously damaged homes or entering general evacuation shelters that were ill-equipped to handle their needs. Stigma often played an important role in these decisions, as one nurse explained:

*“There was a PPD who did not evacuate to a welfare evacuation center because he did not want people to know that he was disabled.”*

*(Participant C)*

For those who did evacuate, the general shelters often exacerbated their distress owing to unsuitable living conditions and a lack of specialized support. This was further complicated by the nature of the emergency aid itself. The constant rotation of unfamiliar support staff, such as disaster psychiatric assistance teams (DPATs), undermined the trust-based care that is fundamental to PPD support, leading to confusion and a reluctance to engage, as explained by one participant:

*“The public health nurses and DPATs changed every week, and users were confused when asked the same questions.”*

*(Participant E)*

### 3.2. Coping Strategies of PVN (Table 3)

#### 3.2.1. Theme 1: Pragmatic Resilience: Phased Resumption and Adaptive Care Delivery

A primary coping strategy demonstrated by PVN was a pragmatic and adaptive approach to service resumption, which carefully balanced the urgent needs of their patients with the severe constraints imposed by the disaster and their own status as victims. Nurses deliberated about how to recommence home visit nursing, prioritizing what was feasible while ensuring their own safety and preventing overwork. This required difficult decisions about resuming duties, as encapsulated by one participant:

*“The nurses were also affected by the disaster, so only those PVN whose domestic situations allowed gathered to discuss resuming work.“*

*(Participant E)*

Adaptations were made to the care delivery process by introducing temporary and flexible measures. PVN reorganized visiting routes and schedules to make visits shorter or more efficient, reflecting a continuous review of how to provide essential care under extraordinary circumstances. One nurse highlighted this necessary flexibility:

*“We visited based on a visit schedule that was more flexible than usual.* *”*

*(Participant F)*

This pragmatic resilience ensured the continued provision of essential care through flexible operational adjustments, safeguarding both patient well-being and caregiver capacity.

#### 3.2.2. Theme 2: Rebuilding the Web of Support: Leveraging Therapeutic Relationships and Systemic Linkages

A core coping strategy for PVN was to actively rebuild the web of support surrounding their patients, which had been fragmented by the disaster. This was achieved through two interconnected approaches: re-establishing personal therapeutic relationships and navigating external systems on behalf of patients. First, participants prioritized restoring a sense of normalcy and security by re-establishing face-to-face contact, leveraging the deep trust they had built with PPDs over time. As one nurse explained, providing routine care was a conscious strategy to mitigate patient anxiety:

*“I thought it would be bad to feel anxious about PPD, so I provided regular home visit nursing care.”*

*(Participant E)*

Second, PVN acted as vital system navigators, using their professional networks to connect PPDs with essential resources that had become inaccessible. This ranged from coordinating with formal disaster response teams to securing medication and new healthcare providers for evacuated patients. A participant described this vital liaison role as follows:

*“Contacted and coordinated with DPAT through the health center with which they already had a connection.* *”*

*(Participant F)*

Through these efforts, PVN acted not just as clinicians, but as the central thread reweaving the torn social and medical safety net for their patients.

#### 3.2.3. Theme 3: A Paradigm Shift to Empowerment: Recognizing Patient Strengths in Crisis

Perhaps the most unexpected finding was a paradigm shift in the nurses’ perception of their patients, from a view focused on vulnerability to one that recognized patients’ inherent strengths and resilience in a crisis. Counter-intuitively, the shared disaster context created opportunities for some PPDs to demonstrate agency and discover new social roles. For individuals who were previously isolated, the structured environment of the evacuation shelter provided a sense of purpose, as one nurse observed:

*“A PPD who felt alienated from those around him before the earthquake was happy to have a role at the evacuation center.”*

*(Participant B)*

This shift also occurred at the community level. The shared experience of the disaster appeared to break down pre-existing social barriers, fostering a new sense of mutual support and understanding between PPDs and other local residents. This led to moments of positive, reciprocal interaction that were less common before the earthquake.

*“At the evacuation center, PPD patients and local residents were considerate toward each other.”*

*(Participant C)*

The recognition of these strengths marked an important cognitive shift for PVN, who changed their purely deficit-based model of care to one that acknowledged the capacity and resilience of PPDs. This shift suggests a more empowering approach to practice.

## 4. Discussion

This study comprehensively elucidated the substantial challenges experienced by PVN in the aftermath of the 2024 NPE. The findings particularly highlight the complexities arising from severe infrastructure damage, the dual role of PVN as both caregivers and victims, and the inherent vulnerabilities of PPDs. Despite these difficulties, PVN demonstrated adaptive and resilient coping strategies that were often rooted in their deep understanding of PPDs and strong community ties. This enabled continuity of care in this crisis setting.

### 4.1. 2024 NPE-Specific Difficulties Experienced by PVN

The Noto Peninsula is a narrow stretch of land that runs along the northern coast of Honshu and has a unique topography. The disruption of major roads and communications in the 2024 NPE was partly a result of the peninsula’s terrain [19,33]. The present findings indicated that PVN found it difficult to confirm the safety of PPDs. The instantaneous and near-total collapse of critical infrastructure [4] created a fundamental barrier to resuming care. A key finding from this study is the exposure of an inherent vulnerability within the community-based care model, which depends on physical access to PPDs’ homes to build trust and provide care. Our observation aligns with previous studies that have identified infrastructural resilience as a prerequisite for continuity of community care in crisis settings [34,35].

The documented effects of the prolonged isolation of the Noto Peninsula are consistent with research on disaster responses in rural and geographically isolated regions. This research consistently reports that infrastructure failure creates “care deserts” that disproportionately affect vulnerable populations reliant on home-based support [36,37]. The implication of this for preparedness is that mental health disaster plans for peninsula-like regions must assume a complete and prolonged loss of conventional infrastructure, necessitating multi-layered strategies such as alternative safety-confirmation methods and training for scenarios where direct access is impossible. After the 2011 Great East Japan Earthquake, electricity was restored to approximately 80% of the affected area (except for Miyagi Prefecture) 3 days after the disaster. In contrast, even 10 days after the 2024 NPE, there was no prospect of power being restored in most areas, mainly because of the peninsula’s topography [38].

Computerized records are now frequently used in many medical institutions. The malfunction of electronic health records and the failure of outdated paper records symbolize an important fragility in information resilience within community psychiatric nursing. This represents a universal challenge for small-scale care providers, who have limited resources compared with large hospitals [39]. This fragility is exacerbated by the person-dependent nature of information management in visiting nursing, in which essential information is often held by individual nurses; this system inevitably collapses when those nurses are themselves disaster victims. This means that the identification of appropriate solutions must move beyond a simple “paper versus cloud” debate. Instead, it is essential to develop a multi-layered information-sharing system. This includes pre-disaster collaboration with local governments and hub hospitals to maintain and share offline-accessible risk information for high-need patients, as well as empowering patients to maintain their own personal health records [40].

Although PVN provide emergency assistance despite such difficulties, an important finding of the present study was that they themselves were also victims in the 2024 NPE.

The difficult situation experienced by the PVN in this study, who were both supporters and victims, has been widely recognized in the field of disaster nursing, which refers to the “dual role” of such personnel. The conflict between their sense of mission and ensuring their own safety is not merely a personal struggle experienced by PVN but a serious dilemma in disaster ethics that has attracted international debate [41]. As previous research highlights [42,43], this state of conflict is directly linked to burnout, poor job performance, and, in the worst cases, professional turnover. This suggests that merely “taking into consideration” the mental burden of supporters is insufficient. Rather, to ensure the continuity of community care, it is essential to integrate practical mental health protection measures with disaster preparedness plans. Such measures should include establishing action guidelines that allow supporters to prioritize their own safety without guilt, and pre-establishing psychological first aid and peer support systems specifically for affected supporters.

The present findings importantly highlight the heightened vulnerability of PPDs in evacuation shelters, where the chaotic environment not only exacerbates their mental health conditions but also exposes substantial gaps in generalized disaster response systems. Even if people with mental health problems are able to evacuate to shelters, their inability to adapt to this unfamiliar setting often leads to symptom deterioration. This vulnerability is exacerbated by systemic flaws in the general shelter system, such as the provision of unsuitable relief supplies. Importantly, in the 2024 NPE, although many PPDs were eligible for entry to specialized welfare shelters, they were unaware of the existence of such shelters, and thus were forced to enter general shelters unable to meet their specific needs. Previous research has shown that PPDs have difficulty living in groups and are prone to becoming unwell in such situations [44,45]. Furthermore, the present findings align with international literature indicating that PPDs may be reluctant to disclose their illness during disaster evacuation, and are therefore less likely to receive the support they need [46]. The exposure to stigma in disaster evacuation centers, as documented in previous disasters [47], not only isolates PPDs but also creates a major barrier to their receiving essential psychological first aid and continuity of care. These findings indicate that a transition is needed from the current one-size-fits-all model of designating general facilities as shelters to an inclusive approach that ensures that vulnerable individuals receive specialized support. This requires establishing a system for providing necessary support through proactive, person-centered planning and training with PPD during normal times, as well as training for shelter staff to mitigate the stigma associated with PPDs.

This study identified a critical failure in the continuity of care for PPDs. The disaster disrupted not only medication supply chains but also the foundation of trust between patients and the healthcare system. The interruption of services from familiar providers and the subsequent disruption of medication supplies forced many PPDs to discontinue their therapy, an important risk factor for relapse. Importantly, participants’ observation that PPDs did not effectively use temporary support from DPATs highlights a key challenge in disaster mental health: the perceived inadequacy of transient, unfamiliar support teams. This finding is in accord with international research emphasizing that for individuals with severe mental illness, the therapeutic alliance is paramount, and trust is not easily transferred to temporary providers [48,49]. The non-apparent nature of many psychiatric conditions further complicates assessment by external teams who lack background knowledge of the patient. This has a major implication for policymaking: ensuring continuity of care [50] in a disaster requires more than just deploying external teams. It requires placing trusted and familiar local providers, such as PVN, at the core of the emergency response to bridge the trust gap and facilitate a “warm handover” to temporary services, thereby ensuring that PPDs receive the care they need.

Natural disasters take a major toll on survivors and their communities [51]. The present study showed how the collapse of community infrastructure creates a profound and distinct crisis for PPDs. The loss of familiar services, businesses, and neighborhood connections, as described by participants, represents a disintegration of the “environmental scaffolding” that is essential for supporting the daily routines and social integration of PPDs. This finding aligns with literature on the social determinants of health and community resilience, which emphasizes that mental well-being is deeply intertwined with community stability [52,53]. As a result of community breakdown, PVN were forced to expand their role beyond clinical care. They effectively became their patients’ primary point of social contact and the main channel for essential information, an important and often unsupported expansion of their professional duties. These findings have major implications for long-term recovery planning and highlight the need to rebuild not only physical infrastructure but also the social and economic fabric essential for a therapeutic community. Additionally, it is necessary to recognize and allocate resources for the non-clinical roles that community mental health nurses inevitably assume in community reconstruction.

### 4.2. The PVNS Strategy in the 2024 NPE

A key strategic finding from this study was the phased and adaptive resumption of services by PVN, which carefully balanced patient care with their own safety and well-being as “dual victims”. This approach transcends a simple restart of operations; it represents a high level of professional judgment and resilience under extreme pressure. This prioritization of caregiver well-being aligns with international principles of disaster nursing ethics, which recognize that the sustainability of the response is dependent on the health of the responders. This suggests that organizational disaster preparedness for community health services must include clear protocols for phased service resumption and robust support systems to protect the mental and physical health of care providers.

The Noto Peninsula’s geographical isolation and the extensive damage to its transportation infrastructure led to substantial delays in external aid [38,54]. As discussed, the trust gap between PPDs and external teams was a major challenge. In response, PVN demonstrated an important coping strategy: leveraging pre-existing local networks and acting as vital liaisons with external emergency teams like DPATs. This positions local providers not merely as passive recipients of aid but as active agents who shared their deep, patient-specific knowledge and used their established community connections to facilitate the overall disaster response. This finding is consistent with international literature on inter-organizational collaboration in disasters, which highlights the critical role of local experts in navigating the complexities of a response [55,56]. A key implication is that national disaster response frameworks, such as Japan’s DPAT system [57,58], must be designed to integrate and empower local providers from the outset, rather than supplanting them.

The present findings reaffirm that the pre-existing, trust-based relationship between PVN and PPDs is a cornerstone of effective disaster mental health response. As indicated by studies on the Japanese PVNS that emphasize interpersonal bonds [59], this therapeutic alliance provided a sense of security for PPDs amidst chaos. This interpretation aligns with the broader principle of “continuity of care” in mental health, suggesting that the therapeutic relationship itself is an essential part of infrastructure that persists even when physical systems collapse. Therefore, long-term investment in community-based mental health services should be viewed as a core aspect of disaster preparedness, as these established relationships are an invaluable and resilient asset in a crisis.

This study highlights the strategic strength of PVN in providing flexible, person-centered support to PPDs who did not or could not evacuate, a known challenge in Japanese disasters [60]. Rather than enforcing a rigid evacuation mandate, PVN adopted a pragmatic, harm-reduction approach, acknowledging patient autonomy while mitigating risks presented by the damage to their homes. In this capacity, they acted as an indispensable information conduit between these hard-to-reach individuals and formal response systems like DPATs. This role is critical, as PVN possess deep, nuanced patient knowledge that external teams lack. This has important implications for disaster policy, suggesting that response plans must include flexible protocols for supporting non-evacuees and formally recognize local providers as strategic partners, not just helpers, in an effective response.

Perhaps the most important finding was the recognition of the unexpected strengths and resilience of PPDs, which challenges the conventional narrative of vulnerability. Contrary to the expectation that the crisis would uniformly worsen symptoms [61], some PPDs exhibited improved daily routines and social connections in shelters, a phenomenon also observed in some contexts during the Great East Japan Earthquake [24]. This finding can be understood with reference to the concept of post-traumatic growth. For some socially isolated individuals, the disaster (although traumatic) served to powerfully break their established patterns of isolation. Simultaneously, the shared communal experience in shelters lowered social barriers and provided new opportunities for connection. This shift from a deficit-based view to a strengths-based view is an important insight and has implications for practice. It suggests that disaster mental health support should not solely focus on mitigating trauma, but must also be designed to identify and cultivate the inherent resilience of PPDs as a foundation for more empowering and effective support.

This study had several limitations. First, it was conducted at one visiting nursing station selected using purposive sampling. Because of the damage caused by the disaster, it was difficult to request research from other visiting nursing stations. The results may have been affected by the organization of this station and may not be applicable to other stations. In addition, this was a post-NPE survey conducted in the unique location of the Noto Peninsula; although it provides important insights into home nursing practice in similar areas, the findings may not be generalizable to urban areas. Moreover, because only one center was included and the number of study participants was small, it is possible that theoretical data saturation was not reached. Furthermore, a general limitation of qualitative research is the limited generalizability of the results. The data analysis did not take into account the diseases or disaster situations of PPDs receiving home nursing care. Additionally, the influence of the authors’ relationships with the participants on the content of the data needs to be considered. Future surveys that include more objective and detailed data are needed.

## 5. Conclusions

This study clarified the difficulties and coping strategies of PVN after the 2024 NPE, providing important insights for post-disaster psychiatric nursing. Regarding policymaking, the findings demonstrate the need to shift disaster response models from a focus on merely deploying external aid to empowering existing, trusted community providers like PVN. The study also highlights the need to formally integrate visiting nursing services into disaster preparedness plans, with protocols that address the “dual role” of caregivers and ensure the resilience of care continuity. Ultimately, these findings advocate a fundamental shift in disaster preparedness, warranting the development of strategies during normal times that are tailored to patient individuality and leverage existing community connections.

## Figures and Tables

**Table 1:** Characteristics of the participants.

Participant ID	Gender	Age	Years of Experience as Nurse	Years of Experience as Psychiatric Nurse	Years of Experience as PVN	Post
A	Male	40–49	23	21	9	Nurse
B	Male	50–59	31	31	3	Nurse
C	Male	50–59	33	33	10	Visiting nurse station manager, Nurse
D	Female	30–39	24	10	5	Nurse
E	Female	50–59	29	29	14	Administrator, Nurse
F	Male	30–39	10	8	4	Nurse

PVN, psychiatric visiting nurses.

**Table 2:** Difficulties experienced by PVN.

Themes	Sub-Themes
Paralysis of care: the collapse of foundational systems	Breakdown of physical access and mobility
	Failure of information and communication
	Collapse of the medical supply chain
The “dual role” dilemma; navigating personal trauma and professional duty	Experiencing disaster as a caregiver
	Ethical and practical prioritization conflicts
Compounded vulnerability; the precarious position of PPDs in a state of exception	Unsafe and unsuitable living conditions
	Systemic gaps in shelter provision
	Fragmented and unfamiliar support systems
	Community disintegration and resource loss

PPDs, persons with psychiatric disabilities; PVN, psychiatric visiting nurses.

**Table 3:** Coping strategies of PVN.

Themes	Sub-Themes
Pragmatic resilience: phased resumption and adaptive care delivery	Prioritizing safety and well-being in service resumption
	Flexible and adaptive redesign of care delivery
Rebuilding the web of support: leveraging therapeutic relationships and systemic linkages	Restoring trust through the therapeutic alliances
	Acting as system navigators and resource brokers
	Providing direct environmental and logistical support
A paradigm shift to empowerment: recognizing patient strengths in crisis	Discovering latent strengths and agency in PPDs
	Observing positive shifts in social dynamics

PPDs, persons with psychiatric disabilities; PVN, psychiatric visiting nurses.

## Data Availability

Dataset is available upon request from the authors. The data are not publicly available due to [privacy and ethical restrictions].

## Public Involvement Statement

There was no public involvement in any aspect of this research.

## Guidelines and Standards Statement

This manuscript was drafted following the Standards for Reporting Qualitative Research (SRQR) for qualitative research.
